# Pressure-Flow Study and Urethrolysis in Treating Female Bladder Outlet Obstruction

**DOI:** 10.7759/cureus.77309

**Published:** 2025-01-12

**Authors:** Nattaporn Wanvimolkul, Patkawat Ramart

**Affiliations:** 1 Division of Urology, Department of Surgery, Faculty of Medicine, Siriraj Hospital, Mahidol University, Bangkok, THA

**Keywords:** female bladder outlet obstruction, fluoroscopic abnormality, periurethral fibrosis, pressure-flow study, urethrolysis, urodynamics criteria

## Abstract

Introduction

Female bladder outlet obstruction (fBOO) is a challenging condition to diagnose. Pressure-flow studies are a key diagnostic tool, but the cutoff remains undefined. This study aims to evaluate the association between pressure-flow parameters and successful outcomes after transvaginal urethrolysis in patients with fluoroscopically confirmed bladder outlet obstruction.

Material and methods

This single-center retrospective cohort study included 30 women who were clinically suspected of having bladder outlet obstruction, with fluoroscopically confirmed bladder outlet abnormalities, and underwent transvaginal urethrolysis. All patients were assessed with a follow-up period of at least six months postoperatively. Success was defined as an improvement in lower urinary tract symptoms and/or the absence of clinical symptoms of cystitis, as reported by patients, along with no pyuria on urinalysis during the follow-up period.

Results

Among the 30 patients included in the study, 14 underwent urethrolysis primarily for clinically suspected bladder outlet obstruction, with a success rate of 64.3% (nine cases). Additionally, 16 patients were assessed for the resolution of recurrent cystitis, achieving a success rate of 50.0% (eight cases). None of the pressure-flow study parameters showed statistically significant differences between the success and failure groups. Postoperative de novo urinary incontinence was reported in 10 cases (52.6%) but none required surgical correction.

Conclusion

No pressure-flow study parameters accurately predict the success rate of transvaginal urethrolysis in treating patients with clinically suspected fBOO and fluoroscopic bladder outlet abnormalities. The success rates of transvaginal urethrolysis were 64.3% for clinically suspected fBOO and 50.0% for recurrent cystitis.

## Introduction

Bladder outlet obstruction in females differs significantly from males due to their simpler anatomy; however, it involves a more complex urination process. According to previous studies, the prevalence of female bladder outlet obstruction (fBOO) can be as high as 29% in specialist centers, while in general hospitals, this condition is often underdiagnosed. This is likely due to the lack of established diagnostic criteria in key investigations such as urodynamics (UDS) or videourodynamics (VUDS) [1].

fBOO can be easily classified into two categories: anatomical and functional problems. Anatomical issues can be further subdivided into intraluminal, intramural, and periurethral origins. In the periurethral group, periurethral fibrosis is a common cause, often associated with recurrent cystitis and lower urinary tract symptoms (LUTSs). This condition is typically caused by chronic inflammation, trauma, and post-anti-incontinence surgery [2]. If left untreated, fBOO may lead to troublesome symptoms, upper urinary tract infections, and even kidney deterioration.

Urethrolysis is a surgical procedure used to lyse periurethral fibrous tissue and restore urethral mobility. The procedure can be performed via transvaginal, suprameatal, or retropubic approach. Previous studies have reported that the success rate of urethrolysis in managing BOO following anti-incontinence surgery and recurrent cystitis is 65% to 87% [3-6] and 53.8% [7], respectively. The variability in success rates is attributed to not only differences in surgical techniques but also the challenges in diagnosing fBOO and the lack of clear criteria for selecting suitable candidates for urethrolysis.

To diagnose fBOO, a thorough clinical evaluation, including a bladder diary, urinalysis, and post-void residual measurements, is required. Additionally, VUDS is widely used as an adjunct to clinical diagnosis for confirmation prior to invasive treatment [8]. Previous studies have attempted to establish definitive diagnostic criteria for fBOO through various methods, including pressure-flow studies (PFSs) [9-11], radiological abnormalities [12], and combinations [13]. Our study aims to determine the cutoff values for PFS parameters in women with fluoroscopically confirmed bladder outlet abnormalities via VUDS and clinically suspected fBOO using outcomes of transvaginal urethrolysis as the reference standard.

## Materials and methods

After receiving approval from the institutional review board, the medical records of 242 female patients who underwent urethrolysis between January 2016 and January 2023 at our institute were retrospectively reviewed.

**Figure 1:**
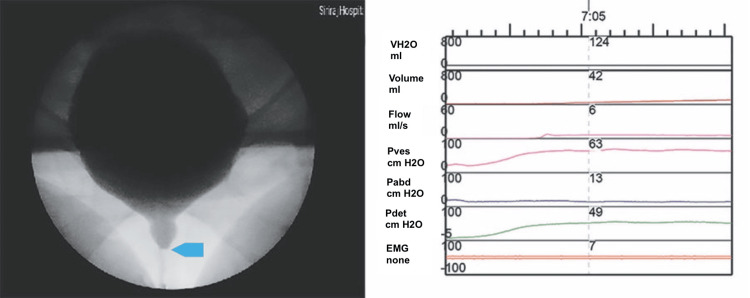
Fluoroscopic findings on VUDS demonstrating urethral disproportion at the midurethra (blue arrow), with a Qmax of 6 mL/sec and PdetQmax of 49 cmH2O. VUDS, videourodynamics

The inclusion criteria comprised cases indicated for urethrolysis due to either clinically suspected fBOO or recurrent cystitis. Clinically suspected fBOO was defined as LUTSs of uncertain etiology, including both storage and voiding symptoms. Recurrent cystitis was defined as experiencing three or more episodes of clinical symptoms, including dysuria, frequency, and urgency or pyuria on urine analysis within the past year. All included cases exhibited a prominent urethra, anterior vaginal scarring, over-angulated urethra, and loss of urethral mobility. Additionally, all must have fluoroscopically confirmed bladder outlet abnormalities on VUDS, characterized by urethral disproportion at any segment (Figure 1). Given that urethrolysis is not yet a universally accepted treatment, patients advised to undergo surgery have failed conservative management, including behavioral therapy, alpha-adrenergic antagonists, and pelvic floor rehabilitation for at least six months. Some patients had also undergone unsuccessful urethral dilation.

The exclusion criteria included pelvic organ prolapse beyond grade 1, a history of pelvic radiation, previous urethrolysis, and incomplete VUDS data. Severe pelvic organ prolapse should be corrected before attributing periurethral fibrosis as the cause of fBOO, while pelvic radiation could degrade the quality of pelvic tissues, complicating the interpretation of the investigation.

Data collected from medical records included age, underlying disease, parity, preoperative LUTS, prior pelvic procedures, and outcomes at a minimum of six months following the procedure. Key PFS parameters were also recorded, including voided volume (VV), post-void residual urine (PVR), maximum flow rate (Qmax), and maximum detrusor pressure at maximum flow rate (PdetQmax). Additionally, the female bladder outlet obstruction index (BOOIf) according to the Solomon-Greenwell formula was calculated [14].

At follow-up (at least six months post-procedure), allowing for the wound healing process, which typically affects urethral and bladder function during the first six months, patients were divided into two groups: success and failure. The definition of success was further subclassified into two categories based on the primary indication. For clinically suspected fBOO, success was defined as an improvement in symptoms, as reported by patients, of more than 80% at the final follow-up. For recurrent cystitis, success was defined as the absence of clinical symptoms and normal urinalysis results at each follow-up visit for the past three months. Additionally, successful cases must not have required any further procedures, including urethral dilation, repeated urethrolysis, or urethroplasty, between six to 24 months post-surgery.

Surgical technique

**Figure 2:**
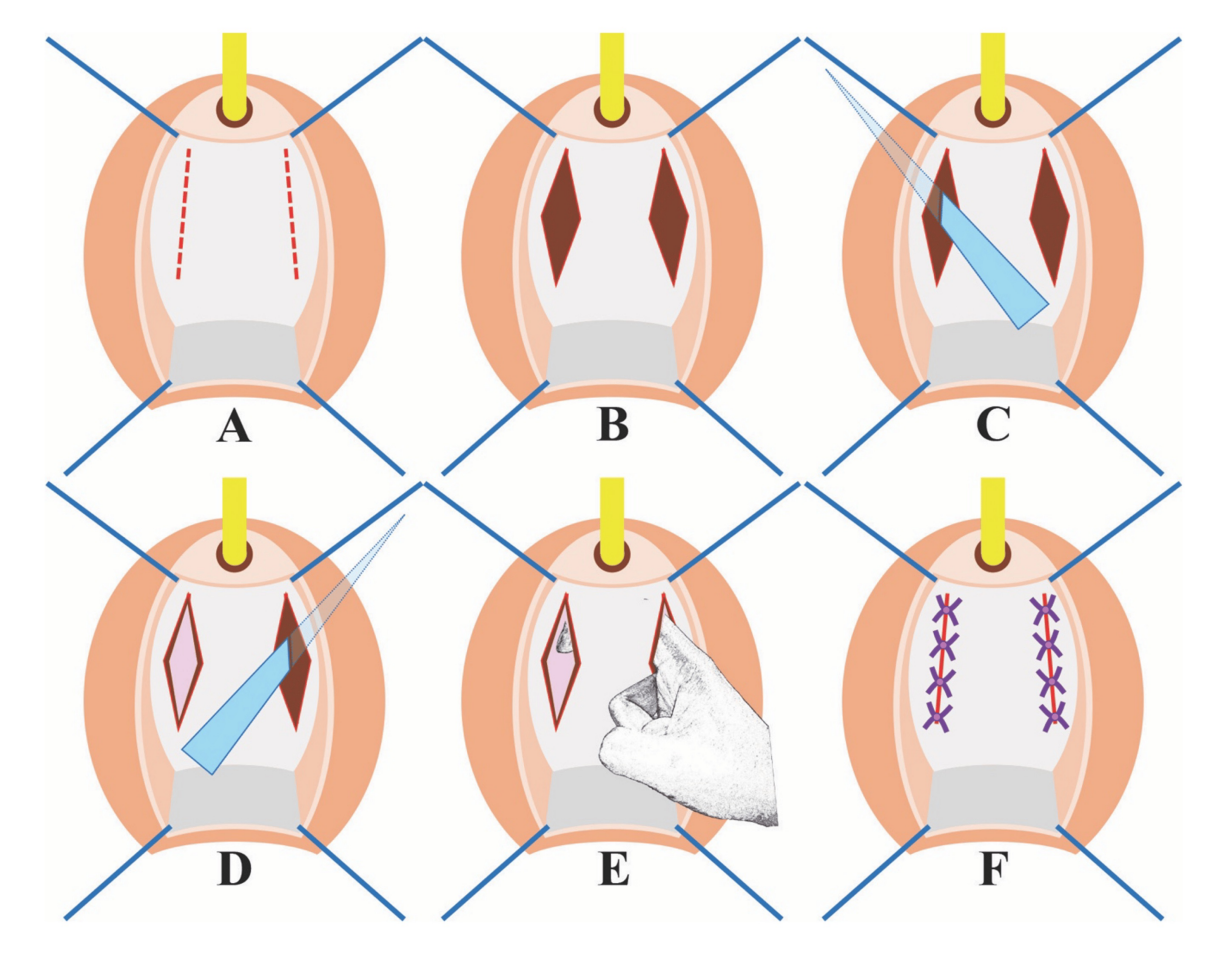
Illustration demonstrating the surgical technique step by step. (A) Dashed lines indicate incisions made at the paraurethral area of the anterior vaginal wall, along the urethra. (B) The pubocervical fascia (in brown) was exposed after dissecting the right vaginal wall. (C and D) A curved mayo scissor was used to puncture and break the fascia on the right and left sides, respectively. (E) Periurethral tissue was bluntly dissected along the urethra by finger until the urethra could be freely pulled downward. (F) The incisions were closed with interrupted stitches using absorbable 2-0 sutures. The illustration was prepared by the author.

**Figure 3:**
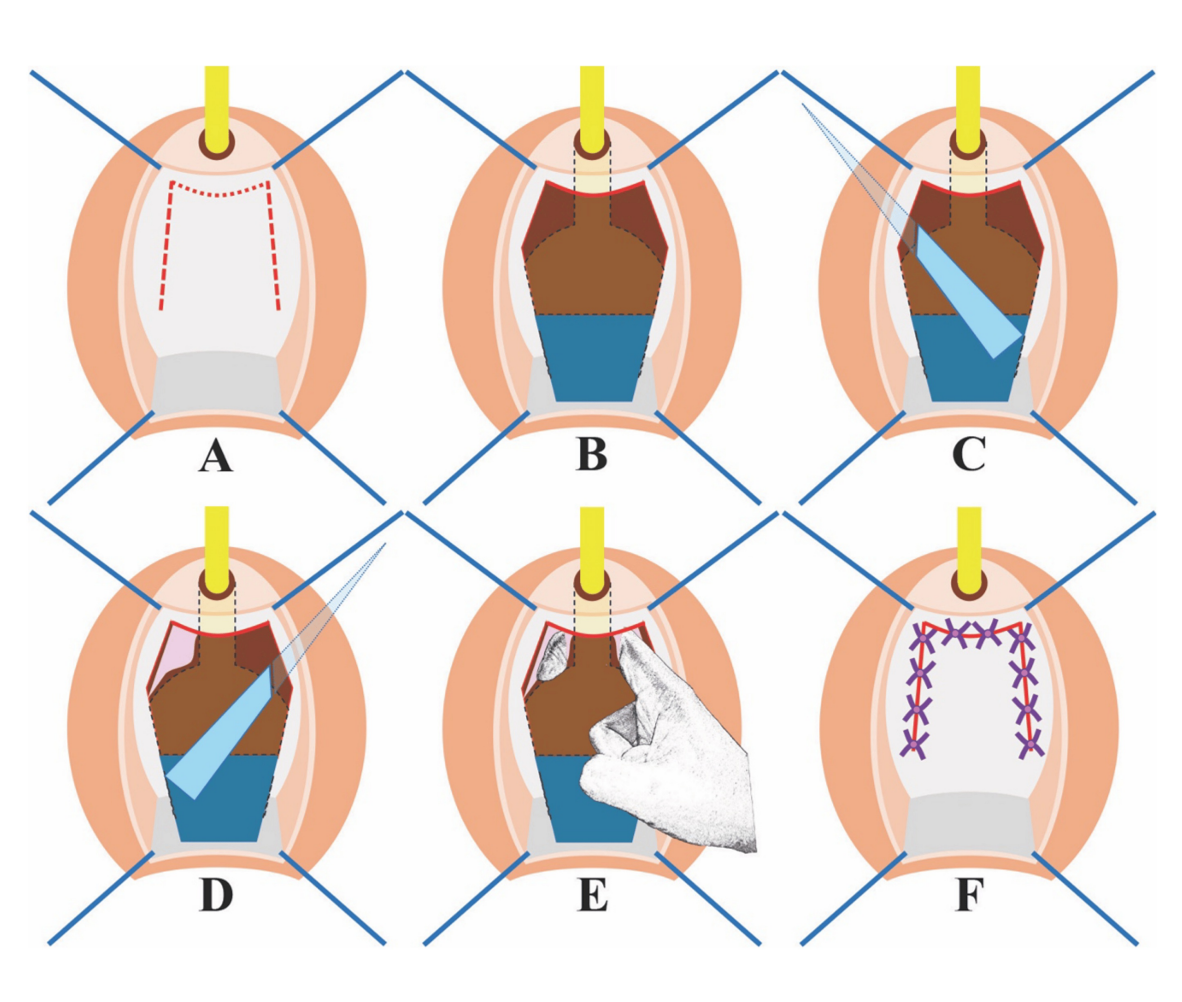
Illustration demonstrating the surgical technique step by step. (A) An inverted U-shaped incision was made on the anterior vaginal wall. (B) The anterior vaginal wall was dissected from the pubocervical fascia, creating a vaginal wall flap. (C and D) A curved mayo scissor was used to puncture and break the fascia on the right and left sides, respectively. (E) The periurethral tissue was bluntly dissected along the urethra by finger until the urethra could be freely pulled downward. (F) The incision was closed with interrupted stiches using absorbable 2-0 sutures after trimming the distal part of the vaginal wall flap to ensure a tension-free closure. The illustration was prepared by the author.

The patient was positioned in an exaggerated lithotomy position. After sterile preparation, the bladder was emptied using a 14 Fr Foley catheter. Two parallel incisions were made along the anterior vaginal wall, following the course of the urethra. The vaginal wall was dissected until the pubocervical fascia was identified. The fascia was punctured with curved Mayo scissors and separated using blunt dissection. The paraurethral and pre-vesical spaces were then bluntly dissected to free the urethra from the surrounding tissue. In some cases, the incision was extended into an inverted U-shape to release and excise distal vaginal wall scarring. A proximal vaginal wall flap was created and pulled down to cover the distal vaginal defect. In cases where urethral mobility was insufficient, as assessed by pulling the urethral catheter, suprameatal urethrolysis was performed by making an additional incision in the suprameatal area. The pubourethral ligaments were detached from the pubic bone. Bleeding was controlled through suturing and packing with gel foam. The incisions were closed with absorbable sutures, and the urethral catheter was maintained for 24 to 48 hours after surgery (Figures 2, 3).

Statistical analysis

Quantitative data, including age and maximum flow rate (Qmax), were reported as means with corresponding minimum and maximum values based on normal distribution. Other VUDS parameters were reported as medians with the minimum and maximum values. Qualitative data, including gender, comorbidities, and LUTS, were presented as frequencies and percentages. The study examined factors influencing the success of transvaginal urethrolysis. Categorical data, including LUTS and underlying diseases, were analyzed using Chi-square or Fisher’s exact tests. Continuous data with normal distribution were analyzed using Student’s t-tests. The Mann-Whitney U test was applied for non-normally distributed continuous data. A regression analysis was performed to assess the impact of VUDS parameters on the outcomes of transvaginal urethrolysis. All analyses were conducted using SPSS Version 18 (IBM Corp., Armonk, NY), with statistical significance defined as a p-value of less than 0.05.

## Results

A total of 242 cases of urethrolysis performed over an eight-year period from January 2016 to January 2024 at our institute were reviewed. Only 30 cases met the inclusion criteria and had complete VUDS with abnormal fluoroscopic findings at the outlet. Of these, the primary indications for transvaginal urethrolysis were clinically suspected fBOO in 14 cases and recurrent cystitis in 16 cases.

The patients' ages ranged from 36 to 81 years, with a mean age of 60.8 years. Among these patients, five had diabetes mellitus, though no diabetic neuropathy was observed. Half of the patients were nulliparous. Seventeen (56.7%) cases had a history of previous pelvic procedures, with some undergoing more than one procedure. These procedures included hysterectomy in nine (30%) cases, urethral dilation in 10 (31.3%) cases, transvaginal vesicovaginal fistula repair in two (6.7%) cases, transvaginal urethral diverticulectomy in one (3.3%) cases, pelvic organ prolapse procedures in one (3.3%) cases, and anti-incontinence procedures in one (3.3%) cases. The most common symptoms in the study group were recurrent cystitis and urgency, occurring in 16 and 14 cases, respectively.

For the PFS parameters in all cases, the mean Qmax was 11.4 mL/sec, the median PdetQmax was 34.3 cmH_2_O, and the mean BOOIf was 4.9.

All outcomes were assessed at a minimum of six months follow-up to ensure tissue healing. In the group where transvaginal urethrolysis was primarily performed for clinically suspected fBOO, 14 cases were evaluated, with a success rate of 64.3% (nine cases). A history of vaginal delivery was the only statistically significant predictor of success (Table 1). In the recurrent cystitis group, 16 cases were evaluated, with a success rate of 50% (eight cases) (Table 2).

**Table 1:** Characteristics associated with success in treating clinically suspected fBOO Non-normally distributed quantitative data are presented as medians with ranges and analyzed using the Mann-Whitney U test. Qualitative data (e.g., age, PFS parameters) are summarized as percentages and analyzed using the chi-square test with exact test. VV, void volume; PVR, postvoid residual volume; Qmax, maximum flow rate; PdetQmax, maximum detrusor pressure at maximum flow rate; BOOIf, bladder outlet obstruction index according to the Solomon-Greenwell formula; PFS, pressure-flow study; LUTS, lower urinary tract symptoms

Characteristics	Total (n =14)	Outcome		Value of the test statistics	p-Value
		Success (n = 9)	Failure (n = 5)		
Age, median (min, max) (years)	62 (36, 81)	65 (55, 81)	45 (36, 67)	0.020	0.020
Diabetes mellitus, n (%)	2 (14.3)	1 (11.1)	1 (20.0)	0.207	1.000
Normal labor, n (%)	6 (42.9)	6 (66.7)	0 (0.0)	5.833	0.031
Cesarean section, n (%)	2 (14.3)	2 (22.2)	0 (0.0)	1.296	0.505
Prior pelvic procedures					
Urethral dilation, n (%)	5 (35.7)	3 (33.3)	2 (40.0)	0.062	1.000
Hysterectomy, n (%)	5 (35.7)	3 (33.3)	2 (40.0)	0.062	1.000
Pelvic organ prolapse procedure, n (%)	1 (7.1)	1 (11.1)	0 (0.0)	0.598	1.000
Transvaginal vesicovaginal fistula repair, n (%)	1 (7.1)	1(11.1)	0 (0.0)	0.027	1.000
Transvaginal urethral diverticulectomy, n (%)	2 (14.3)	1(11.1)	1 (20.0)	0.831	1.000
LUTS before surgery					
Frequency (≥8 times/days), n (%)	3 (21.4)	2 (22.2)	1 (20.0)	0.009	1.000
Nocturia (≥2 times/days), n (%)	9 (64.3)	7 (77.8)	2 (40)	1.998	0.266
Urgency, n (%)	9 (64.3)	6 (66.7)	3 (60.0)	0.062	1.000
Hesitancy, n (%)	4 (28.6)	3 (33.3)	1 (20.0)	0.280	1.000
Straining, n (%)	4 (28.6)	2 (22.2)	2 (40.0)	0.498	0.580
Poor stream, n (%)	3 (21.4)	1 (11.1)	2 (40.0)	1.593	0.505
Sense of residual urine, n (%)	6 (42.9)	4 (44.4)	2 (40)	0.026	1.000
Urgency urinary incontinence, n (%)	7 (50.0)	5 (55.6)	2 (40.0)	0.311	1.000
Stress urinary incontinence, n (%)	3 (21.4)	2 (22.2)	1 (20.0)	0.009	1.000
PFS parameters					
VV, median (min, max) (ml)	281.5 (187, 553)	281.0 (187, 553)	282.0 (189, 465)	-0.200	0.841
PVR, median (min, max) (ml)	5.0 (0, 200)	0.0 (0, 192)	23.0 (0, 200)	-0.641	0.522
Qmax, median (min, max) (ml/sec)	12.6 (1.3, 22)	14 (1.3, 22)	7.5 (6.1, 18)	-1.135	0.298
PdetQmax, median (min, max) (cmH_2_O)	34.2 (11.6, 169.7)	31.8 (13.0, 169.7)	53.4 (11.6, 71.0)	-0.733	0.463
BOOIf, median (min, max)	-3.5 (-29.7, 166.8)	-4.5 (-29.7, 166.8)	37.5 (-17.0, 57.6)	-0.733	0.463

**Table 2:** Characteristics associated with success in treating recurrent cystitis Non-normally distributed quantitative data are presented as medians with ranges and analyzed using the Mann-Whitney U test. Qualitative data (e.g., age, PFS parameters) are summarized as percentages and analyzed using the chi-square tests with exact test. VV, void volume; PVR, postvoid residual volume; Qmax, maximum flow rate; PdetQmax, maximum detrusor pressure at maximum flow rate; BOOIf, bladder outlet obstruction index according to the Solomon-Greenwell formula; PFS, pressure-flow study; LUTS, lower urinary tract symptoms

Characteristics	Total (n = 16)	Outcome			Value of the test statistic		p-Value
		Success (n = 8)	Failure (n = 5)				
Age, median (min, max) (years)	61.4 (42, 71)	62.4 (53, 70)		60.4 (42, 71)		-0.106	0.664
Diabetes mellitus, n (%)	3 (18.8)	1 (12.5)		2 (25.0)		0.410	1.000
Normal labor, n (%)	1 (6.3)	1 (12.5)		0 (0.0)		1.067	1.000
Cesarean section, n (%)	3 (18.8)	0 (0.0)		3 (37.5)		3.692	0.200
Prior pelvic procedures							
Urethral dilation, n (%)	8 (50.0)	5 (62.5)		3 (37.5)		1.000	0.619
Hysterectomy, n (%)	4 (25.0)	1 (12.5)		2 (37.5)		1.333	0.569
Anti-incontinence procedure, n (%)	1 (6.3)	1 (12.5)		0 (0.0)		1.067	1.000
LUTS before surgery							
Frequency (≥8 times/days), n (%)	4 (25.0)	0 (0.0)		4 (50.0)		5.333	0.077
Nocturia (≥2 times/days), n (%)	7 (43.8)	4 (50.0)		3 (37.5)		0.254	1.000
Urgency, n (%)	5 (31.3)	2 (25.0)		3 (37.5)		0.291	1.000
Hesitancy, n (%)	3 (18.8)	2 (25.0)		1 (12.5)		0.410	1.000
Straining, n (%)	4 (25.0)	1 (12.5)		3 (37.5)		1.333	0.569
Poor stream, n (%)	4 (25.0)	3 (37.5)		1 (12.5)		1.333	0.569
Sense of residual urine, n (%)	6 (37.5)	5 (62.5)		1 (12.5)		4.267	0.119
Urgency urinary incontinence, n (%)	2 (12.5)	1 (12.5)		1 (12.5)		0.000	1.000
PFS parameters							
VV, median (min, max) (ml)	209.0 (154, 545)	184.0 (165.3, 429)		288.5 (154, 545)		-0.945	0.345
PVR, median (min, max) (ml)	25.0 (0, 265)	52.5 (0, 265)		0.0 (0, 175)		-0.841	0.400
Qmax, median (min, max) (ml/sec)	9.7 (6.1, 21.5)	9.2 (6.1, 14.2)		10.8 (6.4, 21.5)		-0.840	0.401
PdetQmax, median (min, max) (cmH_2_O)	34.25 (13.8, 86.6)	(20.7, 86.6)		(13.8, 69.0)		-0.840	0.401
BOOIf, median (min, max)	5.87 (-14.1, 72.3)	10.3 (-3.1, 72.3)		2.9 (-14.1, 51.8)		-1.155	0.248

Regression analysis showed that the odds ratios for PFS parameters in predicting the outcomes of transvaginal urethrolysis in treating clinically suspected fBOO and recurrent cystitis were close to 1 and not statistically significant (Tables 3, 4).

**Table 3:** Regression analysis of PFS parameters to predict success of transvaginal urethrolysis for treating clinically suspected fBOO Binary logistic regression was used to analyze data. VV, void volume; PVR, postvoid residual volume; Qmax, maximum flow rate; PdetQmax, maximum detrusor pressure at maximum flow rate; BOOIf, bladder outlet obstruction index according to the Solomon-Greenwell formula; PFS, pressure-flow study

PFS parameters	Odds ratio	95% Confident interval	Wald statistics	p-Value
VV (mL)	0.998	0.987–1.009	0.091	0.763
PVR (mL)	1.003	0.989–1.017	0.170	0.680
Qmax (Ml/sec)	0.903	0.726–1.123	0.838	0.360
PdetQmax (cmH_2_O)	1.001	0.974–1.030	0.011	0.916
BOOIf	1.004	0.982–1.026	0.100	0.751

**Table 4:** Regression analysis of PFS parameters to predict success of transvaginal urethrolysis for treating recurrent cystitis Binary logistic regression was used to analyze data. VV, void volume; PVR, postvoid residual volume; Qmax, maximum flow rate; PdetQmax, maximum detrusor pressure at maximum flow rate; BOOIf, bladder outlet obstruction index according to the Solomon-Greenwell formula; PFS, pressure-flow study

PFS parameters	Odds ratio	95% Confident interval	Wald statistics	p-Value
VV (mL)	0.996	0.986–1.005	0.799	0.371
PVR (mL)	1.006	0.994–1.018	0.851	0.356
Qmax (mL/sec)	0.856	0.647–1.133	1.177	0.278
PdetQmax (cmH_2_O)	1.024	0.965–1.086	0.620	0.431
BOOIf	1.029	0.976–1.085	1.132	0.287

There were no major intraoperative complications, no need for blood transfusion, and no cases of bladder or urethral injuries. Only one patient experienced a low-grade fever, which was successfully treated with antibiotics. Out of 19 patients with preoperative continence, 10 developed de novo urinary incontinence that persisted for more than six months after surgery. Additionally, one patient with preoperative stress urinary incontinence who had not undergone anti-incontinence surgery developed de novo urgency urinary incontinence. However, due to mild symptoms, none of the patients with de novo urinary incontinence required anti-incontinence procedure; symptoms were managed with behavioral therapy, pelvic floor exercises, and medications. In the failure group, three patients underwent redo-transvaginal urethrolysis due to recurrent cystitis. Of these, recurrent cystitis was resolved in two cases.

## Discussion

fBOO is the hidden condition behind various disturbing urination symptoms. It presents diagnostic challenges due to its diverse etiological factors and presentation. Although no specific clinical symptom is directly linked to this condition, some evidence suggests that voiding symptoms may indicate fBOO [15]. Moreover, VUDS is considered a crucial tool for investigation, and there is currently no universally accepted set of criteria for definitively diagnosing this condition [16]. The urodynamic criteria that are used in male BOO are not applicable to fBOO due to different pressure-flow relations [15].

Several studies have historically aimed to establish diagnostic criteria for fBOO using UDS. Chassagne et al. proposed that fBOO could be identified with a Qmax of 15 mL/sec and PdetQmax 20 of cmH_2_O [10]. Nitti et al. suggested that radiographic evidence of obstruction between the bladder neck and distal urethra could aid in diagnosing fBOO [12]. They noted that while there was no absolute cutoff, patients with fBOO typically exhibited higher voiding pressures, lower flow rates, and increased PVR. Lemack and Zimmern suggested that a Qmax of 11 mL/sec and a PdetQmax of 21 cmH_2_O were effective in predicting fBOO, with a sensitivity of 91.5% and a specificity of 73.6% [11]. Blavias and Groutz introduced the BOO nomogram, which combined abnormal fluoroscopic findings and uroflowmetry [13]. Solomon et al. introduced a nomogram and BOOIf, which distinguished fBOO with a sensitivity of 94% and specificity of 93% [14].

Many studies have suggested using both radiological abnormalities of the urethra and PFS to diagnose fBOO. Accordingly, our study included only patients suspected of having fBOO with radiological abnormalities of the bladder outlet and no evidence of pelvic organ prolapse, ensuring the cause was periurethral fibrosis. Transvaginal urethrolysis was used as a standard treatment, as it is believed to help lyse the fibrous tissue around the urethra, thereby restoring its elasticity. We still found that no PFS parameters were able to predict the success of transvaginal urethrolysis in treating clinically suspected fBOO and recurrent cystitis in this specific condition. Additionally, no specific symptoms were associated with its success. However, a history of vaginal delivery emerged as a potential predictor of success in treating clinically suspected fBOO. This association may be due to the significant trauma that vaginal delivery can inflict on the pelvic floor, resulting in subsequent scarring and periurethral fibrosis [17].

To successfully void without experiencing LUTS, the bladder and outlet must work in coordination and maintain balance. This complexity makes achieving normal UDS challenging and highlights the need for further study in female outlet dysfunction.

Our cohort reported a 64.3% success rate for transvaginal urethrolysis in treating clinically suspected fBOO, which is comparable to previous studies. Studies of urethrolysis for treating BOO following anti-incontinence procedures have reported success rates ranging from 65% to 87% [3-6]. This indicated that transvaginal urethrolysis can also be effective in treating fBOO without prior anti-incontinence surgery. In terms of recurrent cystitis, our study demonstrated a 50% success rate, which aligns with the results reported in Boonwong et al.'s study [7].

Potential intraoperative complications of transvaginal urethrolysis were bladder injury and bleeding. Bladder injury could be managed by suturing the injury site and indwelling urethral catheter for two weeks. Bleeding was managed by suturing, cauterization, and applying a hemostatic agent. No cases required reoperation to correct complications. All cases were given oral antibiotics to cover vaginal organisms for at least two weeks.

Urinary incontinence was typically evaluated after six months postoperatively to ensure full tissue healing. Previous studies report urinary incontinence following urethrolysis at rates ranging from 0% to 46.7%, depending on the surgical technique and patient population. In our study, we observed a urinary incontinence rate of 52.6%. All successful cases who reported post-operative urinary incontinence had mild symptoms that did not interfere with their daily life. None of these cases required subsequent anti-incontinence surgery. They all preferred to tolerate some degree of urinary incontinence rather than continue with preoperative LUTSs. The potential complication warrants further investigation to better understand the risk factors and to develop strategies for minimizing its occurrence in patients undergoing this procedure.

The strengths of this study include a comprehensive diagnostic approach that uses physical examination and fluoroscopic abnormalities of the bladder outlet to diagnose fBOO caused by periurethral fibrosis, along with an analysis of variations in PFS. Additionally, the surgical procedure was consistently performed by a single surgeon using the same technique. The limitations of this study included its retrospective design, a small sample size, and potential selection bias based on a wide range of exclusions, which may limit the applicability of the findings to a broader population. Further research is required to identify universal PFS cutoffs for diagnosing fBOO and to ensure the benefits of transvaginal urethrolysis.

## Conclusions

In conclusion, fBOO presents a diagnostic challenge due to the limited and heterogeneous evidence available on diagnostic tests. Transvaginal urethrolysis has emerged as a treatment option for managing fBOO, with a cure rate of 64.3% for clinically suspected fBOO and 50% for recurrent cystitis, respectively. Unfortunately, our study found no PFS parameters reliably associated with the success of transvaginal urethrolysis in treating fBOO.
